# Comment on: Association between the proportion of globally sclerotic glomeruli and various morphologic variables and clinical data of IgA nephropathy patients

**DOI:** 10.12861/jrip.2012.04

**Published:** 2012-01-01

**Authors:** Azar Baradaran

**Affiliations:** ^1^Department of Clinical Pathology, Isfahan University of Medical Sciences, Isfahan, Iran

**Keywords:** Immunoglobulin A nephropathy, Oxford classification, Sclerotic glomeruli

**Implication for health policy/practice/research/medical:**

It is essential to conduct more studies using a larger number of patients or studies from different geographic parts and ethnicities to obtain the other important prognostic morphologic variables, other than four morphologic variables of Oxford classification.

Immunoglobulin A nephropathy is the most common form of primary glomerular disease worldwide (1). Various clinicopathological studies of IgA nephropathy have correlated morphologic variables in diagnostic biopsies with clinical outcomes (1,2). It is noteworthy that, histopathological classifications should be established to predict not only kidney outcomes, but also responsiveness to treatment modalities. The working group of the renal pathology society and international immunoglobulin A nephropathy network proposed the new "Oxford classification" of IgA nephropathy, which consists four pathological variables: mesangial hypercellularity (M0/M1 lesion), segmental glomerulosclerosis (S0/S1 lesion), endocapillary hypercellularity (E0/E1 lesion), and interstitial fibrosis/tubular atrophy (T0/T1/T2 lesion) (1,2). In fact, this classification is an international effort to identify the distinct pathological variables with prognostic value (3,4). This classification is as a split system, which, use semi-quantitative grading of lesion severity in each of the four compartments (glomerular, tubular, interstitial, and vessels compartments) of the kidney and allow the elaboration of a global or aggregate score for each compartment (1-4). However, the Oxford study contained a small number of patients (n=265 patients). Thus, it is essential to conduct more studies using a larger number of patients or studies from different geographic parts and ethnicities to obtain the other important prognostic morphologic variables (2-4). Indeed, after publication of Oxford classification much attempts were made to find other morphologic lesions, which may has prognostic significance. Of them, some pathologic variables, such as extracapillary proliferation (crescent), fibrinoid necrosis of capillary tuft or immunostaining data has been proposed, having prognostic significant (1-4). Nasri *et al*. studied 136 primary IgA nephropathy patients. They aimed to find the relationship of proportion of sclerotic glomeruli with proteinuria and serum creatinine and also with pathologic variables of Oxford classification. They found a significant positive association between proportion of globally sclerotic glomeruli and serum creatinine, amount of proteinuria, and also quantity of tubulointerstitial fibrosis. They also found, significant association of proportion of globally sclerotic glomeruli with M, E, S and T variables of Oxford classification (5). Whether proportion of sclerotic glomeruli has prognostic significance, needs to test in larger studies. However like Nasri *et al*., we also suggest to routinely report of proportion of sclerotic glomeruli in the pathology reports of IgA nephropathy patients.

## Author’s contribution

AB is the single author of the manuscript.

## Conflict of interests

The author declared no competing interests.

## Ethical considerations

Ethical issues (including plagiarism, data fabrication, double publication) have been completely observed by the author.

## Funding/Support

None.
